# Targeted radiopharmaceuticals: an underexplored strategy for ovarian cancer

**DOI:** 10.7150/thno.99782

**Published:** 2024-09-30

**Authors:** Melissa Crabbé, Tomas Opsomer, Koen Vermeulen, Maarten Ooms, Charlotte Segers

**Affiliations:** Nuclear Medical Applications, Belgian Nuclear Research Centre (SCK CEN), Mol, Belgium.

**Keywords:** targeted radionuclide therapy, ovarian cancer, radiopharmaceuticals, theranostics, metastatic disease

## Abstract

Ovarian cancer is the most common gynecological malignancy worldwide with the highest mortality. This low survival rate can be attributed to the fact that symptoms arise only at an advanced disease stage, characterized by a (micro)metastatic spread across the peritoneal cavity. Radiopharmaceuticals, composed of a targeting moiety coupled with either a diagnostic or therapeutic radionuclide, constitute a relatively underexplored theranostic approach that may improve the current standard of care. Efficient patient stratification, follow-up and treatment are several caveats that could be addressed with theranostics to improve patient outcomes. So far, the bulk of research is situated and often halted at the preclinical level, employing murine models of primary and metastatic peritoneal disease that do not necessarily provide an accurate representation of the disease heterogeneity, (intrinsic) drug resistance or the complex physiological interactions with the tumor microenvironment. Radioimmunoconjugates with therapeutic α- and electron-emitting radionuclides have been the prevailing standard, targeting a myriad of cell-membrane markers that are expressed in the various heterogeneous histological subtypes of ovarian cancer. Evidently, several hurdles exist within preclinical research that are potentially withholding these agents from advancing into clinical practice. On the other hand, the field of nuclear medicine has also seen significant innovation to address shortcomings related to target/ligand identification, preclinical research models, radiochemistry, radiopharmacy and dosimetry, as outlined in this review. Altogether, theranostics hold great promise to answer an unmet medical need for ovarian cancer.

## 1. Ovarian cancer: a general introduction

Ovarian cancer (OC) is the fifth leading cause of cancer death in women, with more than 300 000 new cases and over 200 000 deaths in 2020. It mostly affects older, postmenopausal women, with a median age at diagnosis of 63 years 1. In Western Europe, nearly 16 000 new cases and 12 000 mortalities were reported by the GLOBOCAN Global Cancer Observatory in 2020. OC is a highly heterogeneous cancer with about 90% of neoplasms arising from the ovarian surface epithelium. The World Health Organization classification, based on histopathology, immunohistochemistry and molecular analysis, identified at least five distinct subtypes of malignant epithelial OC (eOC), including high-grade serous OC (HGSOC, 70% of cases), endometrioid (10%), clear cell (6 - 10%), low-grade serous (5%) and mucinous (3 - 4%) carcinoma 2. These types strongly differ in origin, pathogenesis, clinical features and prognosis.

The European Society for Medical Oncology recently published a clinical practice guideline for diagnosis, treatment and follow-up of eOC 3. Most women are diagnosed later in life, based on symptoms of which the majority only present at advanced stages (*i.e.* International Federation of Obstetrics and Gynecology [FIGO] stage III and IV), by a combination of pelvic examination, transvaginal ultrasound and serum biomarkers 4. Due to the non-specific nature of the initial symptoms, they may be attributed to non-related pathologies which often causes a delay in the diagnosis. Reported symptoms include abdominal/pelvic pain, constipation, diarrhea, urinary frequency, vaginal bleeding, abdominal distension, fatigue and ascites 3. The late onset of clinical symptoms is characteristic for HGSOC whereas other subtypes may present symptoms at earlier stages of the disease. Although less useful at early stages, combined measurements of serum tumor markers including cancer antigen 125 (CA-125), human epididymis protein 4 (HE4), carcinoembryonic antigen (CEA) and carbohydrate antigen 19-9 (CA-19-9) aid diagnosis 5. Pelvic ultrasound and X-ray computed tomography (CT) of the thorax, abdomen and pelvis complete clinical staging and support surgical planning 5. However, a conclusive diagnosis of OC requires pathological examination of the tumor, lymph node and/or abdominal fluid biopsies.

Once diagnosed, the gold standard treatment for OC involves primary cytoreductive surgery aiming for complete resection, followed by systemic platinum-based chemotherapy 6,7. In addition, angiogenesis (bevacizumab) and poly(ADP-ribose)-polymerase (PARP) inhibitors (olaparib, niraparib and rucaparib) are also considered as maintenance therapy, given their positive results in clinical trials 8-12. Unfortunately, the recommended treatment regimen is only partially successful since up to 70% of patients with stage III - IV HGSOC relapse within three years likely due to acquired chemoresistance 3. The 5-year survival rate for this advanced disease stage is only 30 - 40%. Heterogeneity (*i.e*. intra-tumoral heterogeneity, inter-patient heterogeneity and temporal heterogeneity regarding cancer molecular signature, disease progression, patient's general health and treatment regimen) is considered one of the main obstacles to successful disease management.

Given the important implications for women's health, there is an unmet need for early detection and effective treatment strategies for disseminated cancer to improve the patient's survival and quality of life. To this end, nuclear medicine offers new theranostic opportunities to established or newly identified targets. This review explores the potential of targeted radiopharmaceuticals for eOC patient selection, treatment and follow-up, and their impact on patient care.

## 2. Methods

A systematic search of the Medline (PubMed) electronic database was performed to identify scientific articles, published in press in English between January 1, 2014, and May 31, 2024, that reported on theranostic radiopharmaceuticals in preclinical or clinical research.

The following search terms were used: “ovarian cancer” and “molecular radiotherapy”, “targeted radionuclide therapy”, “targeted radioligand therapy”, “radionuclide” or “radiopharmaceuticals”. References in each scientific article were searched to identify potentially missed studies. Clinical trial registries such as clinicaltrial.gov were examined for active prospective trials using the terms ovarian (cancer) in combination with the various theranostic targets identified from the PubMed electronic library search. The most recent article was selected in case multiple articles were published on the same radioligand by the same authors or research group. We excluded review articles without original data, original research reporting solely on optical probes, clinical case reports or reports from ongoing clinical trials that were not published in a peer-reviewed journal. Section 5, including the tabulated overview, excluded original research with solely diagnostic (imaging) findings.

## 3. Theranostics as a cornerstone for personalized medicine

The principle of theranostics has been the cornerstone of nuclear medicine since the development of radioactive iodine treatment for thyroid disease almost 90 years ago. The approval of [^177^Lu]Lu-DOTATATE (LUTATHERA®) for treatment of neuroendocrine tumors has boosted the demand for theranostic procedures. The concept is centralized around a single targeting moiety that can be conjugated with both imaging radioisotopes for diagnosis, patient stratification and follow-up of disease progression as well as therapeutic isotopes as a treatment to reduce the patient's tumor burden. Incorporated diagnostic radionuclides allow for imaging of primary tumors and metastases using positron emission tomography (PET) or single photon emission computed tomography (SPECT) and selection of patients eligible for subsequent targeted radionuclide therapy (TRT). By performing pretreatment image-based dosimetry, absorbed doses (AD) to tumor tissues and healthy organs can be accurately assessed. This information may facilitate the personalization of administered activities to obtain a higher therapeutic efficacy while minimizing healthy tissue toxicity. Yet, the quest for the optimal combination of target-radioligand remains. In the following sections, we look more closely at radiopharmaceuticals for diagnosis (section 4), therapy and/or theranostics (section 5).

## 4. The current role of radiopharmaceuticals for patient diagnosis and follow-up

Effective screening methods have not yet been implemented in clinical practice, likely due to the relatively low frequency and heterogeneous character of eOC. Known risk factors are germline mutations in the *BRCA1/2* gene or the DNA mismatch repair system 13. Prophylactic salpingo-oophrectomy and long-term surveillance of these patients at risk is highly encouraged. A healthy lifestyle and avoidance of long-term hormone therapy for (post-)menopausal women is also recommended. Nevertheless, early detection of eOC (FIGO stage I; tumor involves one or both ovaries and/or fallopian tubes without pelvic extension) is key in improving survival rates for all patients. Also, reliable preoperative imaging is essential for successful primary cytoreduction given the strong prognostic link between the degree of postoperative residual disease and patients' overall survival (OS) and progression-free survival (PFS) rates 7. CT is currently the best available imaging technique for presurgical evaluation and disease staging 14.

Nevertheless, the current standard practice can benefit from combinations with nuclear imaging. 2-Deoxy-2-[^18^F]fluoro-D-glucose ([^18^F]FDG) PET/CT can provide additional value in differentiating benign from malignant pelvic lesions, as well as tumor, node, metastasis (TNM) staging of OC patients and prognosis 14-16. [^18^F]FDG PET/CT also proved to be a relevant tool for detection of recurrent disease and distant metastases in patients with elevated CA-125 levels 17. The detection of lung recurrence sites and absence of bone lesions, uniquely identified by [^18^F]FDG PET/CT, was shown to be an independent and good prognostic factor 18,19. However, its lack of spatial resolution (~4 mm) results in an underestimation of the involvement of parts of the intestinal tract or mesenteric lymph nodes, in comparison to surgical exploration or magnetic resonance imaging (MRI) 19.

**Figure 1:**
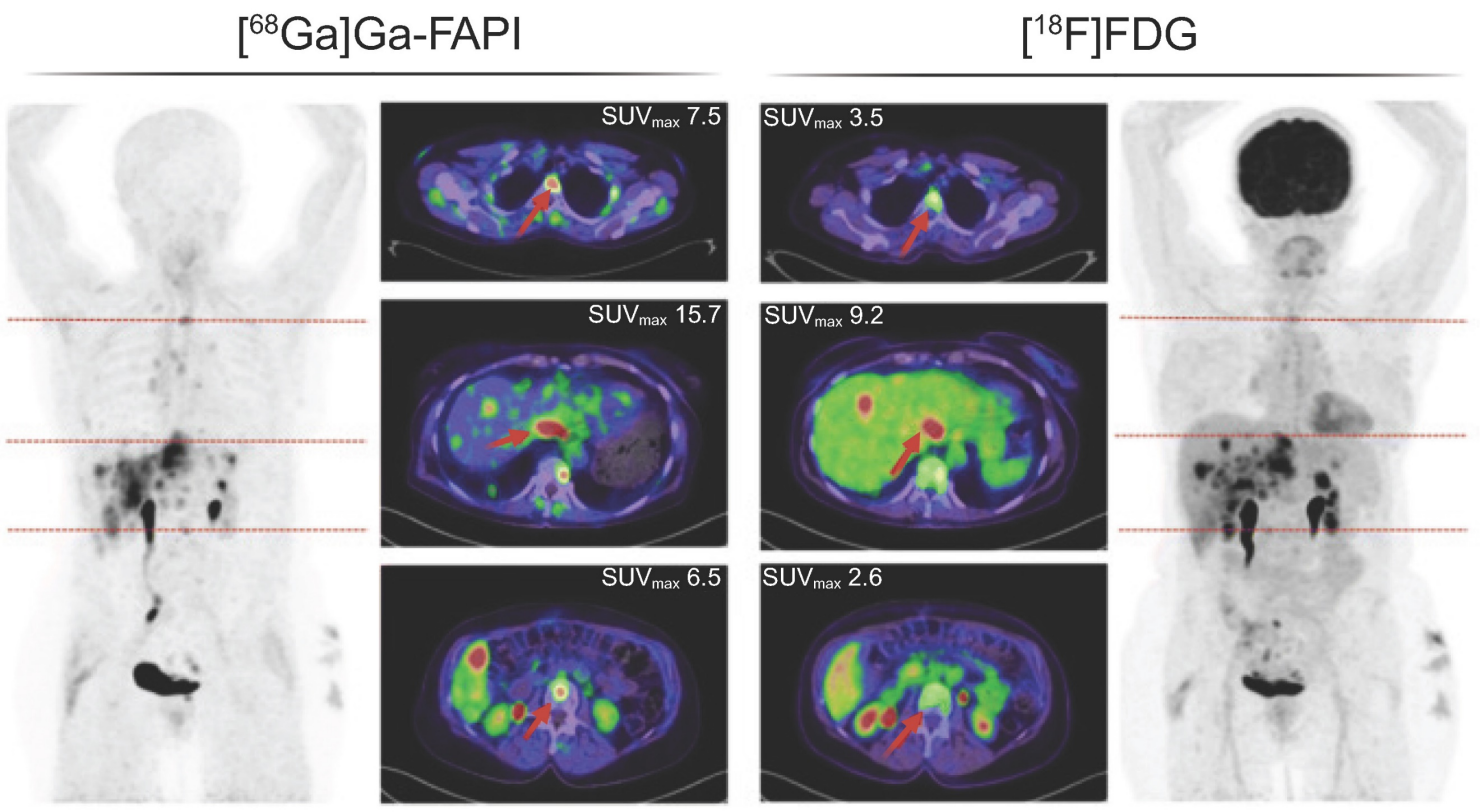
[^68^Ga]Ga-FAPI-PET/CT followed by a [^18^F]FDG-PET/CT one month later in a 63-year-old woman with metastasized ovarian cancer. The normal brain parenchyma and liver depict a clear visual difference in tracer uptake. Adapted with permission from Dendl *et al*. 21 under the Creative Commons Attribution 4.0 International License, © Springer Nature. FAPI: fibroblast activation protein inhibitor; [^18^F]FDG: [^18^F]fluorodeoxyglucose; SUV_max_: maximal standard uptake value.

In the past few years, fibroblast activation protein inhibitor (FAPI) radioligands have emerged as a good alternative for [^18^F]FDG in oncology. In a comparison with [^18^F]FDG PET, [^68^Ga]Ga-FAPI-04 PET/CT reached a higher sensitivity in detection of distant metastases (Figure 1) 20,21. Additionally, [^68^Ga]Ga-FAPI-04 PET/MRI showed superior advantages for diagnosing metastases in the peri-diaphragmatic and gastrointestinal region and prediction of incomplete resectability 22.

Kurata *et al*. also studied [^99m^Tc]Tc-hexakis-2-methoxyisobutyl isonitrile (MIBI) uptake as a tool to detect multidrug resistance (MDR) due to the relation between MIBI uptake and expression of MDR-related and apoptosis-related proteins 23. Other diagnostic PET radioligands such as [^11^C]methionine and 3'deoxy-3-[^18^F]fluorothymidine, relying on inherent characteristics of tumor, *i.e*. increased DNA replication, RNA and protein synthesis, have been investigated in the past but are not routinely implemented for diagnostic purposes 24.

**Figure 2:**
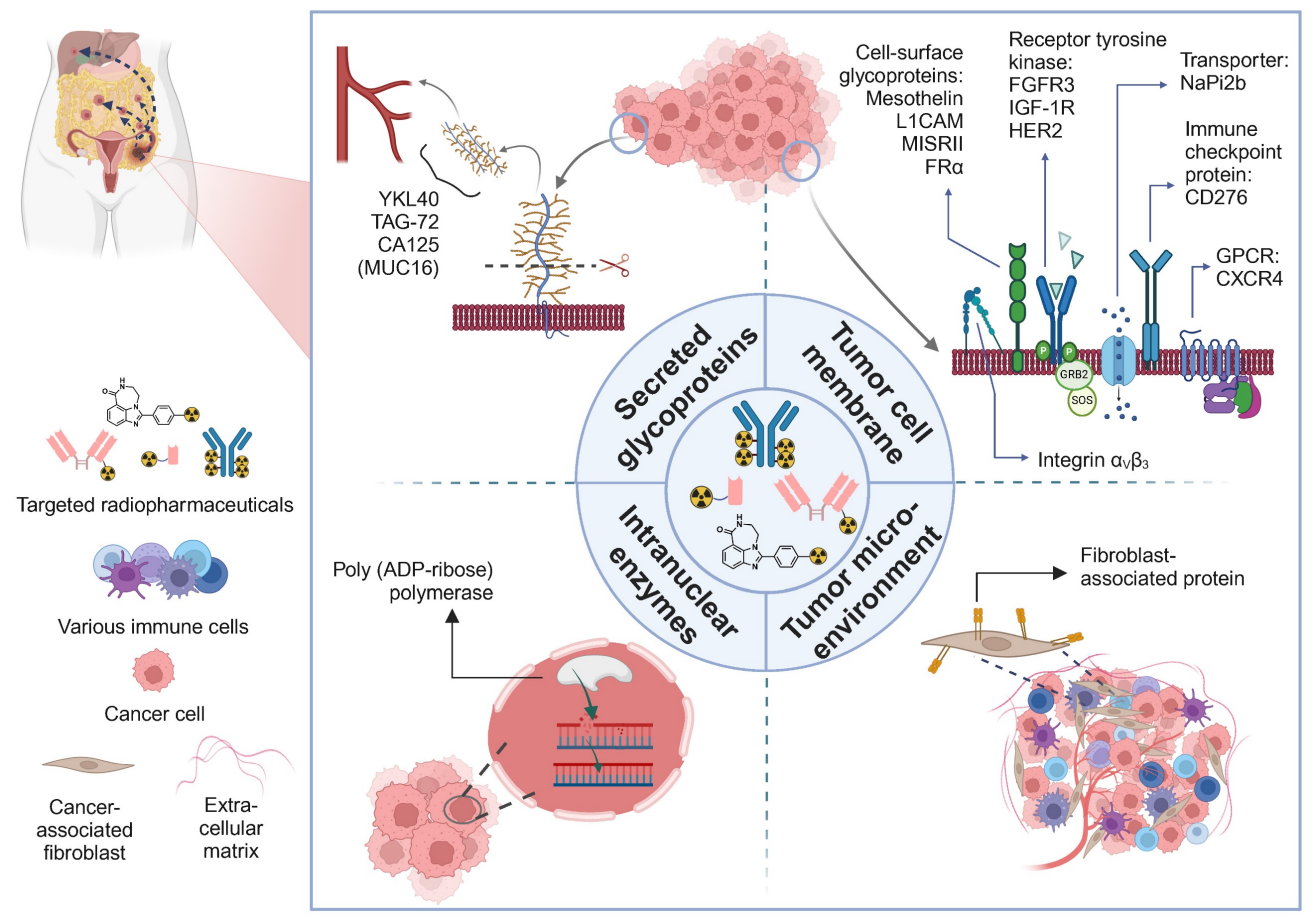
A visual overview of therapeutic radiopharmaceuticals and their targets, utilized in preclinical ovarian cancer models and patients. GPCR: G-protein coupled receptor.

Sadly, besides FAPI radioligands, none of the aforementioned clinical imaging ligands have been proven suitable for theranostic purposes underscoring the need for eOC specific radiopharmaceutical products. To address these shortcomings, several theranostic agents, tailored to the unique molecular characteristics of a patient's tumor, have been emerging. The next section covers an overview of theranostic radiopharmaceuticals, subdivided based on the target localization, *i.e.* circulating in the blood and/or ascites fluid (**5.1**), on the tumor cell membrane (**5.2**), in the nucleus (**5.3**) or within the tumor microenvironment (**5.4**). A visual overview of the theranostic targets studied over the past decade is shown in Figure 2. A tabulated overview is provided in Table 1 (α-emitters) and 2 (electron-emitters).

## 5. Radiopharmaceutical research for ovarian cancer therapy: highlights of the past decade

### 5.1. Glycoproteins and their circulating proteolytic cleavage products

Several research groups have attempted to target glycoproteins such as CA-125 (N-terminal epitope of mucin-16 [MUC16]) 25-27, tumor-associated glycoprotein 72 (TAG72) 28 and chitinase-3-like protein 1, otherwise known as YKL40 29, using radioimmunoconjugates. Upon proteolytic cleavage of transmembrane glycoproteins, their shed (tandem repeat) components circulate in serum and/or ascitic fluid. A downside to this approach entails that most of the AD may not be deposited at the tumor or metastasis site due to these targets' circulating nature unless only the juxtamembrane domain is targeted. Moreover, radiotoxicity of the hematopoietic system is probable and unfavorable when using radioimmunoconjugates targeting the shed forms. Therefore, the approach of targeting such biomolecules, either for diagnosis or therapy, is of lower interest. Instead, targeting the carboxy-terminal portion of these transmembrane glycoproteins, which remains associated with the tumor cells, should warrant further investigation 30,31. Despite its clinical relevance and promising preclinical results, no clinical trials have been initiated yet likely due to an incomplete understanding of the basic cellular processing of MUC16 (*e.g*. cleavage, protein complexity) and low degree of homology between mouse and human MUC16 sequences 32.

### 5.2. Tumor cell membrane targets

A more common approach includes targeting the extracellular domain of cell membrane proteins that are highly expressed on OC cells and/or microenvironment and depict a low expression rate on healthy tissues.

#### B7-H3

The CD276 transmembrane protein, also known as B7-H3, is an immune checkpoint molecule expressed on the surface of tumor, antigen presenting and natural killer cells 33. It may also be expressed as two circulating soluble isoforms in serum and other fluids. Its function is related to intrinsic pro-tumorigenic properties such as proliferation, invasion and metastatic capacity, thereby its expression is not surprisingly correlated with a poor prognosis 34,35. A ^212^Pb-labeled radioimmunoconjugate was shown to significantly enhance the survival of mice harboring ES-2 intraperitoneal (*i.p.*) xenografts by 2 - 2.5-fold (tumor burden was not evaluated) 36. The majority of the *i.p*. injected activity (IA; ~67%) remained in the peritoneum of tumor-bearing mice, next to kidneys, liver and spleen. Interestingly, carboplatin co-treatment led to a synergistic therapeutic effect *in vitro* but not* in vivo*. Due to unfavorable γ-ray emission of lead-212, combination with lead-203 may serve as a matched-pair diagnostic agent.

#### C-X-C motif chemokine receptor 4 (CXCR4)

CXCR4 is a transmembrane G-protein-coupled receptor overexpressed in various solid cancers. In OC, CXCR4 expression increases in parallel with disease stage and metastatic burden, suggesting a role in peritoneal dissemination 37,38. [^68^Ga]Ga-pentixafor and [^177^Lu]Lu-pentixather are clinically implemented theranostic analogs. [^68^Ga]Ga-pentixafor uptake showed the highest SUV_max_ (between 9 - 10) in OC patients compared to other solid tumors and correlated well with immunohistochemistry 39,40. Regrettably, the small sample size limits a definitive conclusion.

#### Folate receptor alpha (FRα)

FRα is a glycosyl-phosphatidyl-inositol-linked cell membrane protein, which internalizes via endocytosis following conjugate receptor binding 41. It has emerged as an interesting tumor target due to its overexpression in ~80% of eOC, including both newly diagnosed and recurrent cases 41,42. Although the occurrence of FRα in normal tissue is limited, kidneys are the most important site of physiological FRα expression and therefore considered a dose-limiting organ 42. Although heavily investigated in various cancers, only one study has been performed in OC over the last decade. [^211^At]At-farletuzumab showed a 6 to 10-fold increase in antitumor efficacy in mice with metastatic disease, in comparison to all control groups, *i.e.* unspecific [^211^At]At-rituximab, unlabeled farletuzumab and PBS 43. However, higher uptake in the pharynx and stomach may indicate accumulation of free astatine-211. Also, despite local *i.p.* administration of the radioimmunoconjugate, a high and long-term uptake in the blood was noted (> 40% IA/g and > 25% IA/g at 3 h and 22 h *p.i.*, respectively).

#### Human epidermal growth factor (HER2)

The most widely explored target is human epidermal growth factor 2 (HER2), a transmembrane receptor tyrosine kinase. A meta-analysis of 34 studies including a total of 5180 OC patients revealed variations in HER2 overexpression in eOC (11 - 66%), whilst low expression was observed in normal ovarian epithelium 44. Despite profound intratumoral heterogeneity, no significant difference was detected between primary tumors and corresponding metastases 45. Either gene amplification or overexpression may lead to aberrant HER2 signaling in OC, and subsequent faster cell growth, DNA damage and increased tumor progression. HER2 overexpression in OC patients was concluded to be an indicator of poor prognosis 44. Although a variety of HER2-targeting therapies (*e.g.* trastuzumab, pertuzumab) have been approved for breast cancer patients, limited successes have been observed in OC patients.

(Pre)clinical research has predominantly been exploring radioimmunoconjugates of trastuzumab and pertuzumab. Local *i.p.* administration of [^212^Pb]Pb-TCMC-trastuzumab appeared safe up to 27.4 MBq/m² in small cohorts of OC patients (*n* = 3-16) with relapsed HER2^+^ peritoneal metastases 46,47. Besides lead-212, trastuzumab was also labeled with mixed α/β-particle emitter lead-214/bismuth-214 eluted from a new ^222^Rn-based generator system for initial preclinical testing 48. Pertuzumab on the other hand is a fully humanized IgG1κ with a shorter biological half-life (T_b_; 10 days) in comparison to trastuzumab (25.5 days). The shorter T_b_ of pertuzumab improved tumor-to-blood ratios following systemic administrations. [^177^Lu]Lu-DOTA-pertuzumab showed enhanced accumulation in SKOV-3 subcutaneously (*s.c.*) xenografted tumors (25.2 ± 1.2% IA/g at 120 h post-injection [*p.i.*]) 49. Still, the long blood retention time of the pertuzumab radioimmunotherapeutic resulted in a moderate uptake in other vital organs, including the liver (estimated human effective dose of 1.2 ± 0.1 × 10^-1^ mSv/MBq) 49. The concept of pretargeting was then introduced to overcome the pharmacokinetic drawbacks associated with large antibodies, as recently reviewed 50. *In vivo* binding of an aminobenzyl-DOTA radiohapten to a bispecific antibody appeared feasible, even for the internalizing HER2 receptor complex 51. An ^225^Ac-labeled bis-DOTA compound, complexed on one side with natural lutetium, was studied for anti-HER2 pretargeted radioimmunotherapy and was only transiently taken up by the tumor (17.33 ± 10.77% IA/g at 1 h* p.i*.) 52. Rapid washout and renal clearance (blood 5.37 ± 1.18 % IA/g and kidneys 5.62 ± 0.95% IA/g at 1 h *p.i.*) resulted in mild renal histopathological changes attributable to radiotoxicity. Besides pretargeting, antibody fragments or engineered antibody formats can be used to enhance tissue penetration.

In the past few years, single-domain antibodies (sdAbs) emerged as a promising new class of vector molecules for TRT. Their fast kinetics and high affinity make them a good candidate for TRT. The first exploration of anti-HER2 sdAbs for TRT of OC was done by D'Huyvetter *et al*. 53. They labeled anti-HER2 2Rs15d sdAbs with lutetium-177 and compared it to [^177^Lu]Lu-DTPA-trastuzumab. Tumor targeting in SKOV-3 bearing mice with [^177^Lu]Lu-DTPA-2Rs15d was 6 times lower compared to [^177^Lu]Lu-DTPA-trastuzumab. However, they observed a spectacular decrease in healthy tissue uptake resulting in higher tumor-to-background ratios. The same sdAb was later labeled with a variety of other therapeutic radionuclides, including astatine-211, iodine-131, bismuth-213 and actinium-225 54-57. Short-lived radionuclides such as astatine-211 and bismuth-213 were used in an attempt to match the T_p_ to the T_b_ of the sdAb. However, these constructs showed only minor tumor uptake (8.6 - 8.9% IA/g at 1 h *p.i.* and 4.9 ± 0.05% IA/g at 15 min *p.i.,* respectively) 54,56. The [^225^Ac]Ac-DOTA-2Rs15d showed higher tumor uptake (9.87 ± 1.38% IA/g at 6 h *p.i*.), which resulted in a higher therapeutic index, when compared to the ^213^Bi-labeled construct (1.0 *vs*. 0.16, respectively) 57. Finally, [^131^I]I-2Rs15d showed high tumor to background ratios and was found to be a promising candidate for HER2^+^ tumors 55. All conjugated sdAbs displayed high-intensity areas in the renal cortex due to their reabsorption. Although kidneys remain the dose-limiting organ for radiolabeled sdAbs, modifications such as megalin/cubulin receptor saturation or chemically/enzyme-cleavable linkers might reduce kidney uptake 58,59. Of note, iso-[^131^I]I-SGMIB-VHH_1028 showed improved tumor uptake and lower kidney accumulation compared to [^131^I]I-SGMIB-2Rs15d which is currently under clinical investigation 60.

#### Integrin α_V_β_3_

Integrins are cell adhesion receptors that are overexpressed in tumoral neovessels and OC cells where it is linked with proliferation, invasion and metastasis of tumors 61. *I.p.* administration of [^64^Cu]Cu-RaftRGD led to superior tumor uptake and inversely correlated with tumor size (1.2 *vs.* 17.2 mm), in comparison to *i.v.* administration, in a mouse model with OC peritoneal metastases 62. An *i.p*. injection of 148 MBq [^64^Cu]Cu-RaftRGD extended survival from roughly 7 to 16 days and significantly reduced ascites.

#### L1 cell adhesion molecule (L1CAM, also known as L1 or CD171)

In eOC, L1CAM is involved in cell proliferation, invasion and migration, which is required for *i.p.* tumor growth and protection from apoptosis 63,64. The extracellular part of L1CAM is subject to membrane-proximal cleavage, generating a ∼200 kDa soluble L1CAM (sL1CAM) which can be detected in the serum and ascetic fluid of patients. sL1CAM has been shown to be a marker for poor progression-free survival and drug resistance 65. While it is clearly involved in pathophysiology of OC, only very little and inconsistent information is available on L1CAM expression in various disease stages. In sequential manuscripts, Lindenblatt *et al*. reported the use of an [^177^Lu]Lu-DOTA-chCE7 radioimmunoconjugate in both *in vitro* and *in vivo* models, in combination with several clinically tested chemotherapy and protein kinase inhibitors 66,67. The combination of 6 MBq [^177^Lu]Lu-DOTA-chCE7 with 31.6 mg/kg paclitaxel synergistically reduced cell viability of IGROV-1 cells and resulted in a significantly extended overall survival (+11 days) in the xenograft model 66. No signs of acute toxicity or weight loss were observed. Paclitaxel was shown to increase radiosensitivity of the IGROV-1 tumors by arresting cells in the G_2_-M cell cycle ~24 h post treatment. Similarly, combination of [^177^Lu]Lu-DOTA-chCE7 with the protein kinase inhibitor MK1775 decreased the IC_50_-values a monumental 14-fold in IGROV-1 cells when applied 48 h post-radioimmunotherapy 67. Combination therapy led to a significantly higher amount of DNA double-strand breaks and increased early-apoptosis. Yet, MK1775 did not have any significant additive effect *in vivo* in comparison with monotherapy. Replacing lutetium-177 with terbium-161 had a superior effect on tumor growth inhibition but also displayed a slightly lower maximal tolerated dose 68.

#### Mesothelin

The mesothelin glycoprotein has been demonstrated to play a role in cell adhesion and metastatic spread, mitigated by its binding to mucin glycoprotein CA-125 69. Mesothelin exhibits a high expression in a number of solid tumors (mesothelioma, ovarian, pancreatic, *a.o*.) and limited expression under physiological conditions in the pericardium and peritoneal/pleural cavities 70. Commercial sponsor Bayer developed [^227^Th]Th-3,2-HOPO-BAY-2287411 to target the membrane glycoprotein mesothelin 71. Nearly complete tumor growth inhibition was observed with 250 kBq/kg and reversible white blood cell suppression indicated a tolerable toxicity profile in an OVCAR-3 xenograft model. The authors do argue that *in vivo* efficacy was highly dependent on the heterogeneity of mesothelin expression, the number of cumulative hits per tumor cell and tumor doubling time. The 3,2-HOPO chelator also appeared suitable for ^89^Zr-based theranostic applications 72. Moreover, combination of [^227^Th]Th-3,2-HOPO-BAY-2287411 with inhibitors of ataxia telangiectasia mutated, ataxia telangiectasia and Rad3-related (ATR) demonstrated synergistic antitumor activity at activity levels that were non-efficacious as monotherapy 73. Despite promising preclinical data, clinical testing appears to have halted at the initial stage (NCT03507452).

#### Müllerian-Inhibiting Substance Receptor type 2 (MISRII)

MISRII, also known as anti-Müllerian hormone type II receptor, is a transmembrane glycoprotein, belonging to the TGF-β family, with its pivotal role related to gonad development and function 74. Consequently, its expression is low in healthy tissues, which is an attractive feature for TRT. MISRII is abundantly expressed in eOC where its ligand was shown to inhibit tumor proliferation both *in vitro* and *in vivo* in transgenic mouse models 75,76. Deshayes *et al*. achieved a 4 - 5 fold higher tumor-to-blood ratio implementing a brief *i.p.* radioimmunotherapy (BIP-RIT) with the humanized antibody 16F12 where [^213^Bi]Bi-16F12 (tumor AD: 3 Gy) outperformed [^177^Lu]Lu-16F12 (tumor AD: 2.5 Gy) in delaying tumor growth 77. BIP-RIT entails the washing of the peritoneal cavity after radioligand injection which significantly improved therapeutic efficacy for both α- and electron-emitters as compared to not-removing the unbound radioactivity. This way, hematological toxicity could be avoided which would allow for an escalation of injected activity to benefit therapeutic efficacy. We do remark that the preclinical model employed, the AN3 CA cell line, is of endometrial adenocarcinoma origin. Hence, it should not be considered as an OC model. A humanized antibody GM102 was granted U.S. Food and Drug Administration (FDA) orphan drug status for treatment of OC but to date no clinical trials have been listed.

#### Sodium-dependent phosphate transporter protein 2b (NaPi2b)

NaPi2b is a member of the SLC34 family of type 2 phosphate transporters and is expressed in the lung, small intestine, salivary glands, liver and kidney 78. Its main role is related to phosphate homeostasis by transporting phosphate through epithelial cells. NaPi2b is (over)expressed in ~80 - 90% of eOC with a particularly high expression in HGSOC 79. Interestingly, neoadjuvant chemotherapy (carboplatin/paclitaxel) downregulated NaPi2b protein expression, but not transcription, and a modest reduction in NaPi2b expression correlated with disease stage in patients 80. NaPi2b preclinical research has mainly focused on α-radioimmunotherapy of minimal residual OC, including astatine-211 and bismuth‑213 linked to murine monoclonal (MX35, 81) or humanized antibodies (Rebmab200 or MX35‑F(ab')2 82,83) to avoid a human anti-mouse antibody response (HAMA). In the OVCAR-3 xenograft mouse model, MX35 and Rebmab200, radiohalogenated by either astatine-211 or iodine-125, showed a similar normal tissue distribution with a high tumor uptake (> 20% IA/g at 24 h *p.i.*) and blood circulation time (> 20% IA/g at 24 h *p.i*.), especially relative to T_p_ of astatine-211 (7.2 h). The theranostic counterpart, [^99m^Tc]Tc-HYNIC-Rebmab200, also showed specific tumor uptake but due to the slow uptake and clearance, an alternative radionuclide with a longer T_p_ may be more suitable in future studies. Treatment with 9 MBq [^213^Bi]Bi-MX35 resulted in growth delay with microscopic tumors present in only 4/18 mice (tumor free fraction: 0.78) at 14 days post-treatment and tumor AD between 25 and 28 Gy 81. In contrast, Frost *et al.* utilized a pretargeting approach with an avidin-conjugated MX35, combined with 1.5 MBq of an ^211^At-labeled effector molecule which proved superior over ^211^At-labeled MX35 alone, especially for microtumors in the millimeter range, where slow penetration of antibodies may limit the AD to the tumor 84. In an OVCAR-3 xenograft model administered with 2.96 and 3.90 MBq, the curative AD was determined between 12.4 and 16.4 Gy, respectively, for tumors < 50 mm^3^ 83. Nonetheless, median survival decreased linearly with the total injected activity between 11-59 weeks, indicative of systemic radiotoxicity. Hereby Bäck *et al*. emphasized the need for long-term toxicity follow-up of α-emitting radioligands.

In a phase 1 study, ^211^At-astatinated murine MX35-F(ab')_2_ fragments with different activities (83 - 355 MBq) were *i.p.* infused in patients in clinical remission and resulted in a favorable biodistribution and no dose-limiting short-term toxicity (largest dose contribution from lungs, stomach and urinary bladder) 85. Similar to the preclinical reports, the authors report the limited specific activity (up to 1 GBq/mg, 86) as a major limitation. Of note, pharmacokinetics of *i.p*. injected radioligands differ between humans *vs*. mice 87. Antibodies have generally a longer retention time in the peritoneum of patients, leading to an improved therapeutic outcome. Nevertheless, using the recommended weighting factor of 20 for α-particles, the effective dose per MBq/L, for a 200 MBq/L administered concentration, of MX35‑F(ab')_2_ would amount to 2.6 Sv, which is associated with a lethal cancer risk of ~10%. MX35-F(ab')_2_ can clearly be administered without acute deterministic radiation toxicities but is associated with a non-negligible long-term stochastic risk.

#### Others

Clinical trials investigating insulin-like growth factor receptor 1 (IGF-1R) and fibroblast growth factor receptor type 3 (FGFR3)-based TRT have been reported to include OC patients (NCT03746431 and NCT05363605 [discontinued Q1 2023], respectively). However, no peer-reviewed articles were published to our knowledge.

### 5.3. (Intra-)nuclear target: poly(ADP-ribose)-polymerase 1 (PARP-1)

Although less successful as a monotherapy, PARP-1 inhibitors have shown promise as adjuvant therapy for OC 9,10,12. To predict and assess the patients' response to such therapy, the prognostic potential of radiotracer [^18^F]FluorThanatrace ([^18^F]FTT) was evaluated *in vitro* 88*.* Indeed, [^18^F]FTT's specific binding ratio correlated with PARP-1 protein expression and to the response of adjuvant PARP-1 inhibitor therapy in OC. Besides its role of prognostic biomarker, nuclear PARP-1 overexpression can also be exploited as a therapeutic target. Radioiodinated inhibitor [^125^I]I-KX1 could target PARP-1 *in vitro* with high affinity (K_d_ = 7.7 nM) 89. Its payload of Auger electrons (AE), delivered in close proximity to the DNA, could induce a dose-dependent increase in γH2AX foci. In addition, its theranostic counterpart [^123^I]I-KX1 showed tumor-specific uptake in OC xenografted mice as observed with µSPECT/CT imaging. The AE-emitting [^77^Br]Br-RD1 also caused significant cytotoxicity, driven by binding site expression and irrespective of *BRCA1* gene expression 90. Still, the EC_50_ (MBq/mL) and D_50_ (Gy) were about a factor 4 - 5 and 3 higher, respectively, compared to [^125^I]I-KX1 for OVCAR-8 cell lines. PARP expression and number of AE emitted per nuclear decay are clearly key factors to the efficacy of AE-emitting PARP inhibitors.

### 5.4. Tumor microenvironment: fibroblast activation protein (FAP)

Cancer-associated fibroblasts (CAF) are an essential component of the tumor microenvironment with protumorigenic effects, such as growth, invasion, metastasis, and treatment resistance 91. Overexpression of FAP on activated CAF within the tumor microenvironment occurs in ~90% of epithelial malignancies, including breast, ovarian, lung and colorectal cancer, supporting FAP as a pan-cancer theranostic target 92. Moreover, FAP is also expressed on the cell membrane of certain OCs 93.

FAPI-based theranostics have not yet been studied in preclinical OC models, to the best of our knowledge. This is likely attributed to the complexity of the tumor microenvironment to be accurately represented. Nevertheless, few treatment studies have included patients with metastasized OC (*n* < 10) under compassionate use. Overall, FAPI treatment was well-tolerated and led to acceptable side effects 94. Small-scale phase 1 studies with [^177^Lu]Lu-FAPI-04, [^177^Lu]Lu-FAPI-46, [^177^Lu]Lu-FAP-2286 and [^90^Y]Y-FAPI-46 demonstrated reasonably low absorbed doses to healthy organs at risk with relatively high uptake in cancer tissue for a wide range of administered activities per cycle: 1.85 - 4.44 GBq [^177^Lu]Lu-FAPI-46 *vs*. 6 GBq [^90^Y]Y-FAPI-46 *vs*. 5.8 ± 2.0 GBq [^177^Lu]Lu-FAP-2286 94-96. Although no follow-up data has been made available, all patients were selected based on pretherapy ^68^Ga-PET scans to ensure adequate tumor uptake. Several clinical trials are currently enrolling patients to test various theranostic FAPI-based ligands in a prospective setting.

Taken together, last decades' radiopharmaceuticals have been primarily targeting tumor cell membrane proteins (B7-H3, CXCR4, FRα, HER2, integrin α_V_β3, L1CAM, mesothelin, MISRII and NaPi2b) besides the (intra-)nuclear target PARP-1 and the FAP-associated tumor microenvironment. Predominantly radioimmunoconjugates were used, although a move towards fast kinetic sdAbs, peptides and small molecules has been observed, as further discussed in the next section.

## 6. Challenges and opportunities for ovarian cancer theranostics, from A to Z

Despite the immense progress in the field of radiopharmaceuticals, several challenges remain, including regulatory hurdles, radionuclide supply chain, cost per treatment round, reimbursement, availability of specialized facilities and access to a skilled workforce to successfully market a theranostic radioligand 97. Also, from a preclinical point of view, several challenges and opportunities were identified.

### 6.1. Target identification

Clearly, a well-recognized target for 'traditional' treatment at pharmacological doses will not necessarily yield an equally effective target for radiopharmaceutical therapy, as nicely outlined by Ting Lee and colleagues 98. Though many oncological targets have been acknowledged for several decades prior to their successful introduction into nuclear medicine (*e.g*. HER2). Recent advances in multi-omics approaches and artificial intelligence as well as surface profiling (for example for antibody, sdAbs and other targeted therapies) are expected to further enhance the identification of biomolecules and signaling pathways linked to cancer development and/or progression 99. Data mining of databases (GEO 100, GTEx 101, SEER 102, ONCOMINE 103, TCGA 104) has also become increasingly popular and accessible (*e.g*. the OC Data Browser for multiomics analyses, 105) despite their limitations regarding race, region and cancer stage captured, and absence of paired healthy tissue data. Recent discoveries include those in the field of miRNAs, epigenetic regulators, immune checkpoint inhibitors, the tumor stem cell compartment and metastatic niche 106,107. Another approach might explore the omics landscape of therapy-induced senescence 108. Although initially thought to be tumor suppressive, recent work has demonstrated that senescence may be detrimental by promoting OC metastasis and invasion, as extensively reviewed by others 108,109.

### 6.2. Radiopharmaceutical considerations

During the radiopharmaceutical development process, each component should be well considered. Clearly, the idea of “one radionuclide fits all” is outdated and various isotopes have been considered for OC. On the diagnostic side, the most extensive clinical work focused on generator- or cyclotron-produced radionuclides such as short-lived fluorine-18, gallium-68, technetium-99m and iodine-123. However, with personalized dosimetry taking a more prominent role in TRT, diagnostic radionuclides with a longer T_p_ (zirconium-89, manganese-52, iodine-124, terbium-155) are required. On the therapeutic side, α- (actinium-225, bismuth-213, astatine-211, lead-212; Table 1) and electron-emitters (lutetium-177, iodine-131, terbium-161; Table 2) have been used with different physical (T_p_, LET, gamma photon yield, etc.) and radiochemical properties (Table 3). Although not yet studied for OC, future research may also include other therapeutic radionuclides (*e.g.* yttrium-90, rhenium-188).

Besides the therapeutic application, the choice of the isotope is influenced by the need to align the T_p_ with the T_b_ of the carrier. Within OC, the majority of therapeutic radioligands tested consist of radioimmunoconjugates (Table 1 & 2). As illustrated within the HER2 section, a typical evolution from antibodies to fragments thereof can be noted.

While antibodies achieve high affinity and selectivity, they suffer from a poor chemical and thermal stability, and their size results in inefficient extravasation, slow kinetics (T_b_ of days/weeks) and low (micro)tumor (< 100-200 µm) penetration. Furthermore, larger molecules (> 45 kDa for globular proteins) may non-specifically accumulate at the target site due to the enhanced permeability and retention effect (EPR). To improve the (micro)tumor penetration of antibodies, Palm *et al*. also debated the use of low-affinity/molar activity antibodies 87,110. The combination of antibodies with deeper tissue penetrating electron-emitters - instead of short range α/electron-emitters - could also improve the microtumor AD, albeit with surrounding healthy tissue damage ​​^87^. Even a combination of isotopes could be beneficial, as observed with [^225^Ac]Ac- and [^177^Lu]Lu-PSMA-617 tandem therapy ​^111^,^112^​.

Researchers have proposed a few concepts to overcome the issue of slow kinetics associated with antibodies. In the case of OC peritoneal metastases, *i.p.* administration has been investigated as an alternative route and is indeed preferred over *i.v.* administration. Alpha-emitters have been employed most frequently to maximize the tumor AD and minimize healthy tissue toxicity when (or if) the conjugates or free isotopes diffuse out of the peritoneum (Table 1). However, with more advanced OC and metastatic spread beyond the peritoneum, systemic administration of radiopharmaceuticals is desired, as already discussed in the HER2 section. Firstly, pretargeting strategies have been attracting attention 113,114. Hereby a tumor-accumulating bispecific or clickable antibody is administered, followed by injection of a rapidly clearing radiolabeled agent that binds the tumor-bound carrier *in vivo* 115​​. However, the immunogenic response to the pretargeting agents by the formation of HAMA or human anti-human antibodies is a known limitation 116. Secondly, engineered antibody fragments, short peptides and small molecules have been receiving considerable interest due to their efficient clearance and tissue penetration 117,118. These carrier types also often show higher thermal and chemical ​stability but may suffer from low tumor re​tention. Dose fractionation would be interesting, as studied with HER2-targeting sdAbs [^213^Bi]Bi-DTPA-2Rs15d and [^225^Ac]Ac-DOTA-2Rs15d 56,57​​. Repetitive administrations of [^225^Ac]Ac-DOTA-2Rs15d prolonged survival, albeit with significant nephrotoxicity ​^57^​. To increase tumor accumulation and/or retention, multiple ligands binding one or more tumor target(s), or serum protein binders can be introduced 119​. ​An example is the increased tumor uptake observed for ^177^Lu-labeled FAP-targeting homodimers ​^120^​. The second generation branched [^177^Lu]Lu-OTAGA.GLU.(FAPi)_2_ even showed faster excretion and lower off-target uptake 121​​, which underlines the importance of the linker structure. Moreover, recent studies with a FAPI-04 derivative and a HER2-targeting sdAb have demonstrated the impact of SuFEx warheads. The use of a phenyl fluorosulfate group for proximity-enabled covalent binding to the target significantly increased tumor uptake and retention while maintaining rapid clearance from healthy tissues 122,123.

Evidently, the way the radionuclide is attached to the carrier molecule also influences the pharmacokinetics. Radiometal chelators, for instance, are primarily designed to offer acceptable kinetic inertness and complexation kinetics but also affect the lipophilicity and charge of the radiopharmaceuticals ​^124^,^125^​. Research has been focusing on finding chelators that allow radiolabeling of heat-sensitive biomolecules, and that enable stable and preferentially site-specific coupling to the carrier without compromising its affinity. In view of a theranostic approach, recent efforts resulted in advanced cyclic (*e.g.* macropa 126, Crown 127,128, TCMC 129, PSC 130, Lumi804^TM^ 131), acyclic (*e.g.* octapa 132, picoopa 133, nonadentate bispidine 134, HOPO-O8/10 135) and hybrid chelators (*e.g.* 3p-C-NETA 136,137) ​^138^.

Even though stable chelation could be achieved, a big point of concern remains the recoil originating from α-particle decay, leading to detachment of the daughter radionuclide from the radiopharmaceutical and potential damage to healthy tissues. This is particularly the case for radium-223, actinium-225 and thorium‑227. Release of the α-emitting bismuth-212 after β-decay of the parent radionuclide lead-212 has also been of concern 139​. However, *i.v.* injected small molecule/peptide radiopharmaceuticals have been reported for which no significant translocation of bismuth-212 was observed in mice 130,140​​. The addition of metal chelators to the formulation has been applied as a strategy to reduce kidney retention ​^36^​. For the ^211^At-based radiopharmaceuticals listed in Table 1, all comprising astatoaryl groups, *in vivo* deastatination is a well-known issue 141. This may result in an increased uptake in stomach, spleen and lungs, as observed with anti-HER2 sdAbs. Non-specific astatine-211 uptake through the sodium iodide symporter can be counteracted with KClO_4_ administration ​^54^. Yet, stability issues are expected to be addressed through the development of alternative radioastatination methods 142.

Next to stable radionuclide incorporation, radiolytic and *in vivo* stability of the ligand are key factors for a successful radiopharmaceutical product. The use of non-natural amino acids, protein PEGylation and cyclization can ameliorate the* in vivo* stability and T_b_ of therapeutic peptides, while cyclization can even enhance cell membrane permeability ​^143^-^145^​. Indeed, the cyclic peptide-based [^177^Lu]Lu-FAP-2286 showed high and sustained tumor uptake and limited side effects in a first-in-man study (including one OC patient *a.o.*) ​^95^.

### 6.3. Study design

The international Atomic Energy Agency (IAEA), European Medicines Agency as well as other organizations have published guidelines related to the study design for (pre)clinical radiopharmaceutical research 14,146,147. From our comprehensive review, it is apparent that few publications have followed these recommendations, which could be attributed to various reasons such as limited resources, a knowledge gap or the research question itself. Even when studies focus on the same target-ligand combination, the study design is highly variable regarding the biological assays used, cell lines, tumor sizes at experimental start, administration routes, time points and even evaluation of therapeutic efficacy for macro- rather than microscopic tumors (Table 1 & 2). Some of these variables, such as tumor volume and cell line have a profound effect on the therapeutic outcome, which has been extensively described for both α- and electron-emitting radionuclides 148,149. The preclinical study design often lacks investigation of toxicity for the typical organs-at-risk (kidney, liver, bone marrow) and is usually limited to the short term follow-up of the animal weight, blood parameters or histopathology of kidney and liver.

#### Preclinical research models

**Figure 3:**
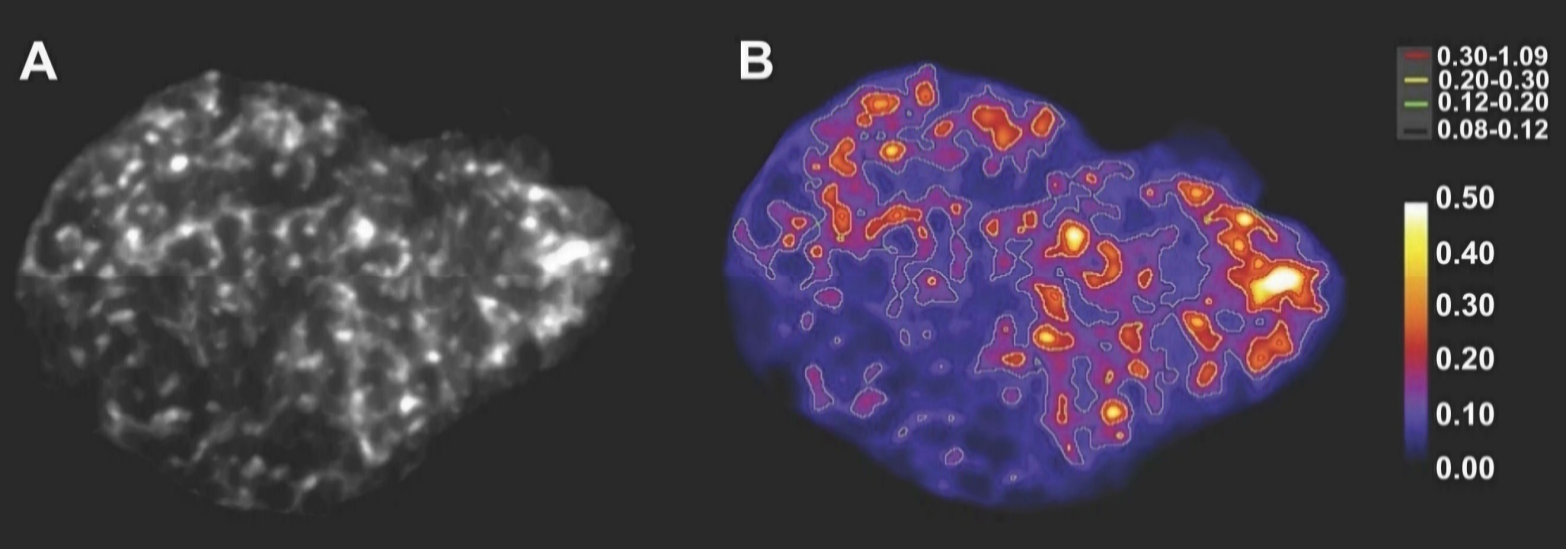
Alpha-camera image of the heterogeneous intra-tumoral activity distribution (A) and dose rate image with isodose curves (in mGy/s/MBq) for different intervals from low (black; 0.08 - 0.12) to high (red; 0.30 - 1.09) (B) of an ^211^At-labeled antibody fragment targeting NaPi2B in an OVCAR-3 tumor slices. This research was originally published in *JNM*. Bäck *et al*. Cure of human ovarian carcinoma solid xenografts by fractionated α-radioimmunotherapy with ^211^At-MX35-F(ab')_2_: influence of absorbed tumor dose and effect on long-term survival. J Nucl Med. 2017;58:598-604. 83 © SNMMI.

A poor concordance between preclinical and clinical studies (in terms of pharmacodynamics and -kinetics but also safety) may be the result of the rather simplistic preclinical research models currently used in the field of nuclear medicine. *In vitro* experiments often rely on monolayers of commercial cell lines (> 80% SKOV-3 & OVCAR-3; Table 1 & 2). Yet, they do not accurately represent the inter-patient and intra-tumor heterogeneity (see Figure 3). Long-term subcultivation negatively impacts the cells' genotype, phenotype and clinical relevance. Regular high-quality cell authentication is thus required. On the other hand, employing transfected cell lines with tremendously higher and homogeneous target expression overestimates the clinical performance of the radiopharmaceutical. Toxicity studies are also hampered by the innate properties of 'healthy' cell lines, which are often immortalized and/or derived from malignancies. A shift is being noted towards the implementation of primary cell cultures, cell co-cultures as well as 3D cell cultures (*i.e.* spheroids, tumoroids, assembloids, patient-derived explants) to simulate *in vivo* conditions. In addition, the choice of an appropriate OC mouse model remains challenging. For now, most theranostics have been investigated in cell-derived *s.c.* or *i.p.* xenografted mice (Table 1 & 2). Better models would include orthotopic engraftment (*i.e.* implantation of tumor cells into the organ or tissue matching the tumor histotype) of patient-derived tumor cells with subsequent metastatic spread into the peritoneum, yet still retaining functional tumor-associated immunocompetent cells (*c.f.* humanized mouse models) 150. These would better capture the complexity encountered in OC patients seeking the need for an appropriate targeted therapy and will improve clinical outcomes. Advances in preclinical OC models have been well-described in recent publications 151-153.

The inherent physiological differences between humans and the preclinical model of choice should also be considered. Differences in peritoneal diffusion rate (for* i.p.* administered pharmaceuticals), human *vs.* murine sequence homology, tumor microenvironment, innate/adaptive immunity and adverse event presentation are just a small number of factors that may affect the successful translation from bench side to bedside.

#### Implementation of combination therapies

As with most malignant tissues, HGSOC thrives on a complex interplay between tumor cells, a stem cell compartment and the tumor microenvironment to shape its outstanding phenotypic plasticity in acquiring resistance 154. A uniform treatment would therefore not be the ideal world scenario to obtain optimal PFS and OS. Yet, preclinical research often focuses on a single treatment regimen which causes a mismatch with the clinical scenario, both regarding standard pretreatment(s) (debulking surgery, chemotherapy, external beam radiotherapy [EBRT]) and adjuvant treatment(s). Many established OC cell lines used for *in vitro* research or xenografted animal models already display chemoresistance possibly due to the original treatment scheme of the diseased host. Kasten and Lindenblatt *et al*. showed a radiosensitizing effect of adjuvant chemotherapy which warrants further research 36,66,67. Garcia-Prada and colleagues demonstrated a radiosensitizing effect of clinically approved gold nanoparticles (for EBRT) in combination with [^177^Lu]Lu-DOTA-trastuzumab in an *i.p.* mouse model 155. Hereby the authors were able to reduce the injected activities while enhancing the therapeutic effect.

To our knowledge, no other adjuvant therapies have been tested for TRT in OC in the last decade. A combination with established (platinum-based) chemotherapy, clinically applied adjuvant therapies (PARP and angiogenic inhibitors) or novel targeted therapies (*a.o.* chimergic antigen receptor T-cells, nanoparticles, immunotherapy, tyrosine kinase inhibitors, oncolytic viruses) could be fruitful for TRT as synergistic effects may lower the required administered activity for tumor control as well as radiation-induced toxicity to healthy tissues 156.

### 6.4. Dosimetry

While in medicine the term dose refers to the mass of the drug to be administered (*e.g.* g), for nuclear medicine the corresponding quantity is activity (*e.g.* Bq). Given that the emission of X- and γ-rays allows the biodistribution of the radiopharmaceutical to be measured quantitatively over time, the administered activity can be converted to the metric of AD (*i.e*. the radiation energy deposited per unit mass [Gy]) for the organs or tissues of interest 157. By taking into account the appropriate radiation weighting factor and the radiosensitivity of the organs, the equivalent and effective dose, can be calculated, respectively.

Still, most of the preclinical studies included in this review (~58%) did not include dosimetry in their study design. On the other hand, almost all clinical trials performed organ-based dosimetry based on the MIRD formalism to derive the maximum tolerated dose (MTD) 158.

Several manuscripts reported limitations within their current dosimetry framework that one should take into consideration. For instance, the radiation weighting factor for α-particles may constitute a too conservative approach for determination of the stochastic effects and may overestimate the true risk to healthy organs given that clinical experience with α-emitters and long-term effects are currently still limited 85,159. In addition, normal tissue dose limits are usually still derived from EBRT but given the differences in radiation toxicity, AD rate, tissue radiosensitivity, heterogeneity of activity distribution and fractionation, dose limits for radiopharmaceuticals may be entirely different and currently lead to underdosing of patients 160. Fractionation may also alter the radiobiological features of the tumors therefore a simple sum of the doses from each fraction may be an oversimplification 83. Due to the inherent heterogeneity within malignant and healthy tissue (Figure 3), the calculation of the mean absorbed doses for organs/tissues are susceptible to errors as, for example, cells on the edges of peritoneal metastases may experience non-specific irradiation from ascitic fluid and experience a higher dose rate, respective to the tumor core, due to a higher antibody binding 81,83. Lastly, the recoil effect may cause a relocation of the daughter isotopes, depending on their half-life, which is difficult to assess with the currently existing technology, but may significantly influence dosimetry 161.

Preclinical studies do offer a wide arsenal of techniques that may address the aforementioned limitations. For example, *in vivo* µSPECT and *ex vivo* digital autoradiography can determine the activity distribution within tumors or organs compartments and even at spheroid/organoid level, to assess dose heterogeneity (Figure 3). Recent advances within *a.o*. DNA damage modeling, computational models for healthy organs and absorbed dose-response models reflect just a glimpse of a rapidly evolving preclinical field 162-164.

## 7. Summary

Not surprisingly, the presentation and detection of HGSOC at advanced stages is inherently tied with a poor prognosis. The diagnosis commonly involves a pelvic examination, CT, ultrasound and/or measurement of serum biomarkers but these techniques lack the sensitivity to detect the disease in a curative stage. Although not implemented in standard practice, nuclear medicine offers both generic diagnostic tools ([^18^F]FDG PET) and tailored theranostics which predominantly consist of radioimmunoconjugates targeting tumor cell-surface proteins. We identified several pitfalls that currently hinder the implementation of theranostics in clinical practice whereas we also highlighted upcoming strategies to overcome these drawbacks.

## Figures and Tables

**Table 1:** Overview of therapeutic and/or theranostic studies with α-particle emitting radiopharmaceuticals in ovarian cancer.

Target	Radioligand	Development stage	Cell line/patient population	Reported radiotoxicity or dosimetry	Summary of (therapeutic) study results	Ref.
**TAG-72**	[^225^Ac]Ac-DOTA-huCC49	Preclinical *in vivo*: *s.c.* xenograft	OVCAR-3	NR	[^225^Ac]Ac-DOTA-huCC49 significantly reduced tumor growth in a dose-dependent manner (1.85, 3.7, and 7.4 kBq), with the 7.4 kBq dose extending survival > 3-fold compared to controls. 1.85 kBq followed by 5 weekly doses of 0.70 kBq for a total of 5.4 kBq extended survival almost 3-fold compared with controls.	28
**B7-H3**	[^212^Pb]Pb-TCMC-376.96 & -TCMC-F3-C25	Preclinical *in vitro*	ES-2 & A2780cp20	NR	Synergistic effect of carboplatin and 2.7 or 21 kBq/mL [^212^Pb]Pb-TCMC-376.96 on clonogenic survival.	36
		Preclinical *in vivo*: *i.p.* xenograft		NR	0.35-0.51 MBq [^212^Pb]Pb-TCMC-376.96 treatment significantly prolonged survival (2 - 3 fold) of mice with *i.p.* tumor xenografts relative to controls. High retention in spleen and liver. No additive effect of carboplatin.	
**FR1α**	[^211^At]At-*m*-MeATE-farletuzumab (& [^211^At]At-MX35)	Preclinical *in vitro*	OVCAR-3	NR	TFF after *i.p.* [^211^At]At-farletuzumab was 91%. Biodistributions revealed accumulation of unlabeled astatine-211 in throat (incl. thyroid) and stomach.	43
		Preclinical *in vivo*: *i.p.* xenograft	OVCAR-3	AD to the nucleus from peritoneal liquid and cell membrane was 7.6 Gy and 9.6 Gy, respectively.	TFF of control groups ranged from 9% to 14%. TFF after [^211^At]At-farletuzumab was 91%.	
**HER2**	[^212^Pb]Pb-TCMC-trastuzumab	Clinical: phase 1 trial	3 patients with HER2^+^ ovarian malignancies and disease progression	No evident short- or long-term toxicity upon follow-up over > 6 months	First-in-human experience with *i.p*. infusion of [^212^Pb]Pb-TCMC-trastuzumab (7.4 MBq/m^2^). No redistribution out of peritoneal cavity.	46
		Clinical: phase 1 trial	18 patients with HER2^+^ peritoneal metastases	No late toxicity (renal, liver, cardiac or other) < 1 year after treatment	*I.p.* [^212^Pb]Pb-TCMC-trastuzumab up to 27 MBq/m² appears safe for patients with peritoneal carcinomatosis who have failed standard therapies. Serum TAG-72 levels better correlated to imaging changes in OC patients than the tumor marker, CA125.	47
	[^214^Pb]Pb- & [^214^Bi]Bi-TCMC-trastuzumab	Preclinical *in vitro*	SKOV-3 & OVCAR-3	NR	0.37 MBq/well reduced clonogenic survival more than 4-fold.	48
		Preclinical *in vivo*: *i.p.* xenograft	SKOV-3	NR	Fractionation (2 x 0.74 MBq) was more efficient compared to single *i.p*. administration (0.74 MBq) in reducing tumor mass (> 5 fold for both treatment schedules).	
		Preclinical *in vivo*: *s.c.* xenograft	SKOV-3	NR	Sustained tumor retention of the Ab until 120 h* p.i.* (~25% IA/g). Blood clearance within 120 h.	
	[^211^At]At-SAGMB-2Rs15d, -SAB-2Rs15d & -MSB-2Rs15d	Preclinical *in vitro*	SKOV-3	NR	Highest specific binding for [^211^At]At-SAGMB-2Rs15d (~66%) and [^211^At]At-MSB-2Rs15d (~77%) after 1 h incubation.	54
		Preclinical *in vivo*: *s.c.* xenograft	SKOV-3	Highest AD to tumor (2 Gy/MBq) and kidneys (7.7 Gy/MBq) for [^211^At]At-SAGMB-2Rs15d was also associated with best therapeutic window. Astatinated sdAbs with *m*-MeATE or MSB reagents indicated the presence of released astatine-211 in lungs/stomach.	Comparable tumor uptake in all radioconjugates (> 8% IA/g at 1 h). [^211^At]At-SAGMB-2Rs15d showed minor uptake in normal tissues. Astatinated sdAbs consisting of *m*-MeATE or MSB reagents revealed elevated uptake in lungs and stomach, indicating free astatine-211. α-camera imaging revealed a homogeneous tumor activity distribution. Fast washout into urine (~3 h *p.i.*).	
	[^213^Bi]Bi-DTPA-2Rs15d	Preclinical *in vitro*	SKOV-3	NR	Clonogenic ability, cell growth rates and cellular apoptosis were significantly impacted upon treatment with [^213^Bi]Bi-DTPA-2Rs15d.	56
		Preclinical *in vivo*: *s.c.* xenograft	SKOV-3	An activity escalation study up to 2 - 11 MBq induced signs of toxicity in kidneys and spleen. Co-infusion of gelofusine significantly reduced kidney uptake.	Administration of [^213^Bi]Bi-DTPA-2Rs15d alone and in combination with trastuzumab resulted in a significant increase in median survival.	
	[^225^Ac]Ac-DOTA-2Rs15d	Preclinical *in vitro*	SKOV-3	NR	There was no treatment-specific effect on colony formation and DSB formation *in vitro*.	57
		Preclinical in vivo:* s.c*. &* i.p*. xenograft	SKOV-3	Mild to serious tubulopathy in mice treated with [^225^Ac]Ac-DOTA-2Rs15d resulting in AD ~9.8 - 29.5 Gy (inflammatory lesions and tubular dilation).	Dose fractionation (3 x 85 kBq) was more efficient compared to a single dose to prolong survival (~factor 3 compared to controls).	
**Mesothelin**	[^227^Th]Th-BAY 2287411	Preclinical *in vitro*	OVCAR-3 and ST103	NR	[^227^Th]Th-BAY 2287411 induces significant cytoxicity by causing DNA DSB, G2-M cycle arrest and ROS production.	71
		Preclinical *in vivo*: xenograft and PDX models	OVCAR-3 and ST103	NR	Strong correlation between MSLN expression levels and tumor uptake. Tumor accumulation close to 100% IA/g at 672 h in the ST103 model. Complete tumor remission with single dose of 500 kBq/kg of [^227^Th]Th-BAY 2287411 in ST103 and near-complete tumor response with 250 kBq/kg for OVCAR-3 *in vivo*.	
	[^227^Th]Th-BAY 2287411	Preclinical *in vitro*	OVCAR-3/8	NR	ATRi and PARPi potentiate [^227^Th]Th-BAY 2287411 therapy by suppressing DNA damage repair.	73
		Preclinical *in vivo*: *s.c.* xenograft	OVCAR-3/8	NR	Enhanced therapeutic efficacy of [^227^Th]Th-BAY 2287411 in combination with ATRi or PARPi in OVCAR-3 and OVCAR-8	
**NaPi2b**	[^213^Bi]Bi-MX35	Preclinical *in vivo*: *i.p.* xenograft	OVCAR-3	No significant differences in platelet or WBC count at 6 and 14 days post treatment. Tumor AD between ~ 11.3 Gy and 27 Gy for 3 and 9 MBq, respectively.	78% of mice were in remission (no macroscopic or microscopic tumors) after single *i.p*. administration of 9 MBq [^213^Bi]Bi-MX35	81
	[^211^At]At-MX35-F(ab′)2	Preclinical *in vivo*: *s.c.* xenograft	OVCAR-3	Bone marrow recovery was noted for the low-activity groups, whereas for high-activity groups the reduction was close to acute myelotoxicity. Decreased hematocrit was seen at a late interval (34 - 59 weeks after therapy).	Complete remission is achievable for < 50 mm^3^ tumors. Complete remission (TFF, 100 %) was found for tumor AD of 12.4 and 16.4 Gy.	83
	[^211^At]At-MX35-F(ab′)2	Clinical: phase 1 trial	6 patients in clinical remission from recurrence	No acute or deterministic radiation toxicities up to 297 MBq. The urinary bladder, thyroid, and kidneys (1.9, 1.8, and 1.7 mGy per MBq/L) received the highest AD.	Absolute activity in the blood peaked at ~12 h (~3% of infusate activity).	110
	[^211^At]At-MX35-F(ab′)2	Clinical: phase 1 trial	12 patients with relapsed epithelial ovarian cancer	Escalation to 355 MBq without dose-limiting toxicities in patients (median follow-up time: 42 months). No decreased tolerance to relapse therapy,	Overall median survival was 35 months, with a 1-, 2-, 5-, and 10-year survival of 100%, 83%, 50%, and 25%, respectively. Lower SA is associated with a lower single-cell dose, whereas a high SA may result in a lower central AD in microtumors. Individual differences in AD to possible microtumors were due to variations in administered activity and the SA.	85

%IA/g: % injected activity/gram of tissue; Ab: antibody; AD: absorbed dose; ATRi: ataxia telangiectasia and Rad3-related protein inhibitor; DSB: double-strand break; *i.p*.: intraperitoneal; KO: knock-out; sdAb: single-domain antibody fragments; NR: not reported; PARPi: poly (ADP-ribose) polymerase inhibitor; PDX: patient-derived xenograft; *p.i*.: post-injection; ROS: reactive oxygen species; SA: specific activity; *s.c*.: subcutaneous; TFF: tumor free fraction.

**Table 2:** Overview of therapeutic and/or theranostic studies with electron-emitting radiopharmaceuticals in ovarian cancer.

Target	Radioligand	Development stage	Cell line	Reported radiotoxicity or dosimetry	Summary of (therapeutic) study results	Ref.
**YKL40**	[^111^In]In & [^177^Lu]Lu-DTPA-YKL40/c41 or /c24	Preclinical *in vivo*: *s.c.* xenograft	CA5171 & ES-2	Significant body weight loss and hematuria. No toxic features in liver, spleen, lungs and kidneys H&E sections.	Significant tumor volume reduction with 7.4 to 22.2 MBq of [^177^Lu]Lu-DTPA-YKL40/c41. Higher therapeutic effect for lutetium-177 *vs*. indium-111.	29
**HER2**	[^177^Lu]Lu-DOTA-trastuzumab	Preclinical *in vivo*: *i.p.* xenograft	SKOV-3 & OVCAR-3	Exposure of Gd-NPs to lutetium-177 increased the AE yield but not the AD.	5 MBq of [^177^Lu]Lu-DOTA-trastuzumab in combination with 2 x 5 mg Gd-NPs resulted in the highest tumor mass reduction.	155
	[^177^Lu]Lu-DOTA-pertuzumab	Preclinical *in vitro*	SKOV-3	NR	Specific binding up to 24% in SKOV3 cells with 65 - 70% internalization.	49
		Preclinical *in vivo*: *s.c.* xenograft	SKOV-3	NR	Sustained tumor retention of the Ab until 120 h* p.i.* (~25% IA/g). Blood clearance within 120 h.	
	[^177^Lu]Lu & [^111^In]In-DTPA-2Rs15d	Preclinical *in vivo*: *s.c.* xenograft	SKOV-3	Equivalent AD in the tumor and kidneys (0.9 Gy/Mbq), 5x lower than [^177^Lu]Lu-DTPA-trastuzumab. No radiotoxicity was observed.	Highest tumor uptake was observed at 1 h *p.i.* ~6.5% IA/g *vs*. kidney 10.4% IA/g. Significantly longer event-free survival for ^177^Lu-treated mice (> day 125) *vs.* controls (day 33 - 75).	53
	[^131^I]I-SGMIB-2Rs15d	Preclinical *in vitro*	SKOV-3	NR	The cell-associated fraction remained stable over 24 h with an internalized fraction up to 50%.	55
		Preclinical *in vivo*: *i.p.* xenograft	SKOV-3	Highest AD to tumor (11.9 Gy) *vs*. kidneys receiving 9.4 Gy. Effective dose in humans was estimated at 0.0273 mSv/MBq. No radiotoxicity was observed.	Fast renal clearance was observed (< 0.5% IA/cc after 4 h) with relatively low tumor uptake (~2% IA/cc). [^131^I]I-SGMIB-2Rs15d treatment prolonged survival by 36% but with high inter-animal variability.	
	*Iso*-[^131^I]I-SGMIB-VHH_1028	Preclinical *in vivo: s.c.* xenograft	SKOV-3	Tumors received a 2.9 higher cumulative AD compared to [^131^I]I-SGMIB-2Rs15d, resulting in an 0.148 mSv/MBq effective dose in humans.	A single administration between 10 - 56 MBq significantly delayed tumor growth compared to control but not between different activity levels.	60
**L1CAM**	[^177^Lu]Lu-DOTA-chCE7	Preclinical *in vitro*	IGROV-1	NR	[^177^Lu]Lu-DOTA-chCE7 in combination with paclitaxel (24 h prior to radioligand) significantly decreased cell viability and increased radiosensitivity *in vitro* in a synergistic manner.	66
		Preclinical *in vivo*: *s.c.* xenograft	IGROV-1	NR	*In vivo* combination therapy of [^177^Lu]Lu-DOTA-chCE7 and paclitaxel (24 h after radioligand) resulted in ~2-fold prolonged overall survival compared to monotherapy. Paclitaxel did not influence radioligand biodistribution 72 h *p.i*.	
	[^161^Tb]Tb-DOTA-chCE7 & [^177^Lu]Lu-DOTA-chCE7	Preclinical *in vivo*: *s.c.* xenograft	IGROV-1	Higher acute radiotoxicity for ^161^Tb-labeled chCE7 (MTD: 10 MBq) compared to lutetium-177 (MTD: 12 MBq).	[^177^Lu]Lu- and [^161^Tb]Tb-DOTA-chCE7 showed comparable high tumor uptake (37.8 - 39.0% IA/g, day 6) with low uptake in healthy organs. For equitoxic doses, tumor growth inhibition was better by 82.6% for the ^161^Tb- *vs.* ^177^Lu-labeled ligand.	68
	[^177^Lu]Lu-DOTA-chCE7	Preclinical *in vitro*	SKOV-3 extracted from ascitic fluid + IGROV-1	NR	Administration of protein kinase inhibitor MK1775 after or together with [^177^Lu]Lu-DOTA-chCE7 administration (0.05 - 5 MBq/mL) increased radiosensitivity and apoptosis* in vitro*.	67
		Preclinical *in vivo*: *s.c.* xenograft	SKOV-3 extracted from ascitic fluid	NR	MK1775 showed no additive effect on therapeutic efficacy of 6 MBq [^177^Lu]Lu-DOTA-chCE7 *in vivo*.	
**MISRII**	[^177^Lu]Lu-DOTA-16F12, [^213^Bi]Bi-DTPA-16F12, [^89^Zr]Zr-DFOM-16F12	Preclinical *in vivo*: *i.p.* xenograft	AN3-CA	Hematologic toxicity was more pronounced with [^177^Lu]Lu-16F12 than with [^213^Bi]Bi-16F12 for *i.p.* injections	*I.p.* treatment with [^177^Lu]Lu-16F12 was slightly more efficient in delaying tumor growth than [^213^Bi]Bi-16F12. Conversely, bismuth-213 was significantly more efficient than lutetium-177 when the peritoneal cavity is washed to remove unbound radioactivity.	77
**Integrin α_V_β_3_**	[^64^Cu]Cu-cyclam-RAFT-c(-RGDfK-)4	Preclinical in vivo:* s.c*. &* i.p*. xenograft	OVCAR-3 & IGROV-1	Kidney was dose-limiting organ (24.6 Gy). Only minor and recoverable hematological toxicity was observed until 60 days *p.i*.	[^64^Cu]Cu-RaftRGD showed an inverse relationship between uptake/therapeutic efficacy and tumor size. Intratumoral heterogeneity linked regions of RaftRGD uptake to sites of α_V_β_3_-positive cancerous cells, angiogenesis and hypoxia.	62
**PARP1**	[^125^I]I-KX1, [^123^I]I-KX1 & [^131^I]I-KX1	Preclinical *in vitro*	OVCAR-8-wt, OVCAR-8^-PARP1 KO^, SKOV-3, SNU251, UWB1.289-BRCA1^mut & restored^	Leftward shift in dose-response curves for [^125^I]I-KX1, compared to [^131^I]I-KX1 in HRD cells. This shift was PARP1-specific. Average RBE ~3 with lowest value in BRCA1 mutant ovarian cancers.	[^125^I]I-KX1 caused a dose-dependent increase in γH2AX foci that was PARP-1 specific at 0.925-3.7 MBq/mL.	89
		Preclinical *in vivo*: *s.c.* xenograft	OVCAR-8	NR	[^125^I]I-KX1 increased expression of yH2AX (ns) in patient tumor slices.	
	[^77^Br]Br-RD1 & [^76^Br]Br-RD1	Preclinical *in vitro*	murine ID8, OVCAR-8^-wt & PARP1 KO^, UWB1.289, UWB1.289-BRCA1^mut & restored^	NR	PARP-expression dependence of [^77^Br]Br-RD1 radiotoxicity is driven by differences in specific binding site expression, in which the loss of PARP1 did not change the radiosensitivity of the cancer cell line. [^77^Br]Br-RD1 cytotoxicity was independent of *BRCA1* gene expression.	90
		Preclinical *in vivo*: healthy mice	-	Bone marrow was the dose-limiting organ, limiting the clinical IA at ~110 GBq.	A clear discrepancy was noted between *in vivo* and* ex vivo* biodistribution, related to heterogeneous uptake and blood/enteric content.	

%IA/g: % injected activity/gram of tissue; AD: absorbed dose; HRD: homologous recombination DNA repair deficiency*;* H&E: hematoxylin & eosin*; i.p*.: intraperitoneal; KO: knock-out; MTD: maximal tolerated dose; NPs: nanoparticles; NR: not reported; ns: not significant; OS: overall survival; PARP: poly (ADP-ribose) polymerase; *p.i*.: post-injection; *s.c*.: subcutaneous; wt: wild-type.

**Table 3:** Physical and chemical properties of most frequently used therapeutic radionuclides for targeted radionuclide therapy of ovarian cancer.

	electron-emitters		α-emitters			
Radionuclide	^177^Lu	^161^Tb	^225^Ac	^211^At		^212^Pb
(and daughters)			(^221^Fr/ ^217^At/ ^213^Bi)	(^211^Po/ ^207^Bi)		(^212^Bi/ ^212^Po/^ 208^TI)
						
T_p_ (d)	6.64	6.95	9.92	0.3		0.44
						
Decay mode (%)	β^-^ (100)	β^-^ (100)	α (100)	ε (58.2) & α (41.8)		β^-^ (100)
(and daughters)			α (100)/ α (99.99)/	α (100)/ ε (100)		β^-^ (64.06) & α (35.94) / α (64.05)
			β^-^ (97.8) & α (2.2)			β^-^ (35.94)
						
Principal E_β/α_, keV (%)	148.8 (79.4)	157.4 (65)	^225^Ac: 5830 (50.7)	^211^At: IC: 78.5 (47) & AE: 8.5 (105)		^212^Pb: 93.28 (81.5) & IC: 148.1 (31.0)
	AE: 6.2 (8.6)	AE: 5.2 (87.9)	^221^Fr: 6341.0 (83.3)	^211^Po: 7450 (98.9)		^212^Bi: 6340 (35) & 6300 (26)
	IC: 101.7 (6.8)	IC: 39.9 (42.4) & 16.6 (41)	^217^At: 7066.9 (99.9)			^212^Po: 10180 (42)
			^213^Bi: 491.8 (66.8)			^208^TI: 649.5 (49.1) & 441.5 (24.2)
						
Principal E_γ/XR,_ keV (%)	208 (11)	25.6 (23.2)	^221^Fr: 218.0 (11.4)	^211^At: 81.5 (29) & 78.9 (18)		^212^Pb: 238.6 (43.6) & 77.1 (16.4)
	113 (6.6)	48.9 (17.7)	^213^Bi: 440.5 (25.9)			^208^TI: 583.2 (85.0) & 510.8 (22.6)
		74.6 (10.3)				
						
Principle particle range in tissue (µm)	280 (β^-^)	301 (β^-^), 13 (IC), 0.1 (AE)	^225^Ac: 47 (α)	60 (α)		^212^Po: 50.1 (α)
			^213^Bi/^213^Po: 85 (α)			^212^Bi: 91.0 (α)
						
Oxidation state	3+	3+	3+	1-/ 1+ *		2+
						
Conventional chelator(s)	DOTA/CHX-A"-DTPA	DOTA/DOTAGA	DOTA/Macropa	NA; astatoaryl compounds		DOTA/TCMC
						
Theranostic pair	^111^In/ ^68^Ga/ ^89^Zr/ ^64^Cu	^152^Tb/ ^155^Tb	^111^In/ ^68^Ga/ ^226^Ac/ ^132^La/ ^133^La	^209^At/ ^131^I/ ^124^I		^203^Pb

Data was extracted from the Evaluated Nuclear Structure Data File (ENSDF) database, 165-171. AE: Auger electron; IC: internal conversion electron; NA: not applicable; T_p_: physical half-life; XR: X-ray; * most common_._

## Abbreviations

%IA/g: % injected activity/gram of tissue
Ab: antibody
AD: absorbed dose
AE: Auger electrons
ATR(i): ataxia telangiectasia mutated, ataxia telangiectasia and Rad3-related (inhibitor)
BIP-RIT: brief intraperitoneal radioimmunotherapy
BRCA1/2: breast cancer gene 1/2
CA-125: cancer antigen 125
CA 19-9: carbohydrate antigen 19-9
CAF: cancer-associated fibroblasts
CEA: carcinoembryonic antigen
CT: X-ray computed tomography
CXCR4: C-X-C motif chemokine receptor 4
DNA: deoxyribonucleic acid
DSB: double-strand break
EBRT: external beam radiotherapy
eOC: epithelial ovarian cancer
EPR: enhanced permeability and retention
FAP: fibroblast activation protein
FAPI: fibroblast activation protein inhibitor
FDA: U.S. food and drug administration
[^18^F]FDG: [^18^F]fluorodeoxyglucose
FGFR3: fibroblast growth factor receptor type3
FIGO: International federation of obstetrics and gynecology
FRα: folate receptor α
GEO: gene expression omnibus database
GTEx: genotype-tissue expression project
HAMA: human anti-mouse antibodies
H&E: hematoxylin & eosin
HE4: human epididymis protein 4
HER2: human epidermal growth factor 2
HGSOC: high-grade serous ovarian cancer
HPLC: high-performance liquid chromatography
HRD: homologous recombination DNA repair deficiency
IA: injected activity
IAEA: International atomic energy agency
IGF-1R: insulin-like growth factor receptor 1
i.p.: intraperitoneal
KO: knock-out
L1CAM: L1 cell adhesion molecule
LET: linear energy transfer
MDR: multidrug resistance
MTD: maximum tolerated dose
MIBI: [^99m^Tc]Tc-hexakis-2-methoxyisobutyl isonitrile
miRNA: micro ribonucleic acid
MIRSRII: Müllerian-inhibiting substance receptor type 2
MRI: magnetic resonance imaging
MTD: maximal tolerated dose
MUC16: mucin-16
NaPi2B: sodium-dependent phosphate transporter protein
NPs: nanoparticles
NR: not reported
ns: not significant
OC: ovarian cancer
OS: overall survival
PARP: poly(ADP-ribose)-polymerase
PDX: patient-derived xenograft
PET: positron emission tomography
PFS: progression-free survival
p.i.: post-injection
PSMA: prostate-specific membrane antigen
ROS: reactive oxygen species
SA: specific activity
s.c.: subcutaneous
sdAb: single-domain antibody
SEER: surveillance, epidemiology, and end results program
sL1CAM: soluble L1 cell adhesion molecule
SPECT: single photon emission computed tomography
SUV: standard uptake value
TAG-72: tumor-associated glycoprotein 72
T_b_: biological half-life
TCGA: the cancer genome atlas
TFF: tumor free fraction
TGF-β: tumor growth factor β
TNM: tumor, node, metastasis
T_p_: physical half-life
TRT: targeted radionuclide therapy
wt: wild-type
